# Evaluation of plasma activated liquids for the elimination of mixed species biofilms within endoscopic working channels

**DOI:** 10.1038/s41598-024-79276-4

**Published:** 2024-11-19

**Authors:** Naomi Northage, Vasyl Shvalya, Martina Modic, Thorsten Juergens, Sascha Eschborn, Malcolm J. Horsburgh, James L. Walsh

**Affiliations:** 1 Centre for Plasma Microbiology, Department of Electrical Engineering and Electronics, University of Liverpool, Liverpool, L69 3GJ UK; 2 Laboratory for Gaseous Electronics, Jožef Stefan Institute, Ljubljana, 1000 Slovenia; 3 R&D Endoscopy Reprocessing Systems, Olympus Surgical Technologies Europe, Olympus Winter & Ibe GmbH, Kuehnstraße 61, 22045 Hamburg, Germany; 4 Infection Biology & Microbiomes, Institute of Infection, Veterinary and Ecological Sciences, University of Liverpool, Liverpool, L69 7BE UK; 5 York Plasma Institute, School of Physics, Engineering & technology, University of York, York, YO10 5DQ UK

**Keywords:** Cold atmospheric plasma, Plasma activated liquids, Reactive oxygen and nitrogen species, Flexible endoscopes, Endoscope reprocessing, High level disinfection., Biofilms, Colonoscopy, Plasma physics

## Abstract

The use of reusable flexible endoscopes has increased dramatically over the past decade, however despite improvements in endoscope reprocessing, the continued emergence of endoscopy-associated outbreaks as a result of multi-drug resistant bacteria has highlighted the need for a new approach to disinfection. Here, the use of plasma activated liquids (PALs) for the elimination of mixed species biofilm contamination within the working channels of endoscopes was evaluated. Cold atmospheric pressure plasma was used to chemically activate water and a commercially available pH buffered peracetic acid to create PALs. Polytetrafluoroethylene endoscope surrogate test pieces were contaminated with clinically relevant mixed species biofilms. The efficacy of PALs for the decontamination of narrow lumens was compared against the commercial disinfectant. Plasma activation was found to increase the antibiofilm capabilities of pH buffered peracetic acid by introducing reactive chemical species into the solution. Disinfection of endoscopic test pieces with plasma activated disinfectant (PAD) resulted in a 7.30 log_10_ reduction of biofilm contamination in 5 min, surpassing the 4.39 log_10_ reduction observed with the currently used endoscope disinfection method. PAD also resulted in reduced regrowth and recolonization of the surface of the endoscopic test pieces. Minimal changes to the surface morphology and composition were observed following exposure to PAD in comparison to the commercial disinfectant, suggesting the developed approach is no more aggressive than current disinfection approaches.

**Supplementary Information:**

The online version contains supplementary material available at 10.1038/s41598-024-79276-4.

## Introduction

Reusable flexible endoscopes have become a prevalent feature of modern medicine, with approximately 2 million endoscopic procedures performed annually in the UK^1,2^. Despite the significant increase in use over the past decade, it has been reported that the rate of healthcare-associated infections resulting from endoscope cross-contamination is 1 in every 1.8 million procedures^3,4^. However, this figure is considered to be a significant underestimation^3^. There has been a lack of detailed surveillance for infections following endoscopy, gross underreporting, and a lack of recognition of acknowledged transmissions^5^. The continued emergence of multidrug-resistant organisms and their involvement in endoscopy-associated outbreaks highlights the significance of endoscopy transmission events and the need for increased transparency^3^. It has been suggested that 91% of post-endoscopy associated infections could have been prevented if quality control systems were improved, emphasizing the need for improvements^6,7^. The two leading causes of patient cross-contamination are inadequate decontamination procedures, which lead to the formation of build-up biofilms within the endoscope working channels, and ultimately equipment malfunction^4,8^.

The complex nature of flexible endoscopes and their repeated exposure to mucous membranes provide ideal conditions for the accumulation of organic material and biofilm growth within the inner channels^8,9^. Following clinical use, endoscopes undergo reprocessing, a multistep, time-consuming procedure involving manual cleaning, leak testing, high level disinfection (HLD), rinsing, drying, and storage^10^. Automated endoscope reprocessors (AERs) are commonly used to minimise contamination and reduce user contact with chemicals, however manual cleaning methods are still necessary and contamination can still occur without failures in the process^11^.

Peracetic acid is a commonly used disinfectant because it is an oxidising agent with action against a wide range of pathogenic bacteria, viruses, and spores^12–14^. It is effective at low temperatures; however, it can be corrosive, depending on its pH and concentration^15^. Furthermore, peracetic acid has been observed to cause fixation of biofilms, resulting in limited efficacy in biofilm removal^15^. Damage to the inner channels of flexible endoscopes can further increase the likelihood of biofilm formation and contribute to reprocessing failures, resulting in partial killing and regrowth of microbes^16^. Methods of sterilization, such as the application of ethylene oxide, have previously been applied in endoscope reprocessing to overcome the issues mentioned, however these have been shown to result in damage and a shortened lifespan of flexible endoscopes^17,18^.

Cold atmospheric pressure plasma (CAP) is a promising technology that has proven useful in many healthcare related applications, including microbial disinfection and surface modification^19–21^. Recent advancements in the area of CAP have shown that the cocktail of reactive chemical species generated exhibits antimicrobial properties against a large array of pathogens, both in planktonic and biofilm states^20^. It is evident from prior studies reported within the literature that CAP processes offer several advantages as a method of high-level disinfection (HLD) over currently adopted methods, with evidence of efficient bacterial inactivation within short timeframes, lower associated costs, and reduced likelihood of toxicity to personnel due to the short half-life of the reactive species^22,23^. However, gas disinfection methods, like CAP or ethylene oxide, typically exhibit reduced efficiency when organic debris and biofilms are present, poor penetration power, and an inability to reach all areas of the complex devices^18^.

Plasma activated liquids (PALs) offer a method of disinfection that may overcome issues observed with gas-phase CAP disinfection. The treatment of liquid with CAP creates a solution rich in reactive oxygen and nitrogen species (RONS), typically reducing the pH and introducing significant antimicrobial activity^24,25^. Prominent studies have focused on the activation of water, referred to as plasma activated water (PAW), and shown its efficacy against various microbes^24–26^. While there are some studies detailing the use of plasma to eliminate biofilm contamination in the context of endoscopy, research on the use of PALs is limited^22,23^. Through this investigation, the application of PALs for the elimination of mixed species biofilm contamination within endoscopic working channel test pieces was explored and compared to a widely used and commercially available disinfectant.

## Materials and methods

### Plasma device and liquid activation

**Fig. 1:**
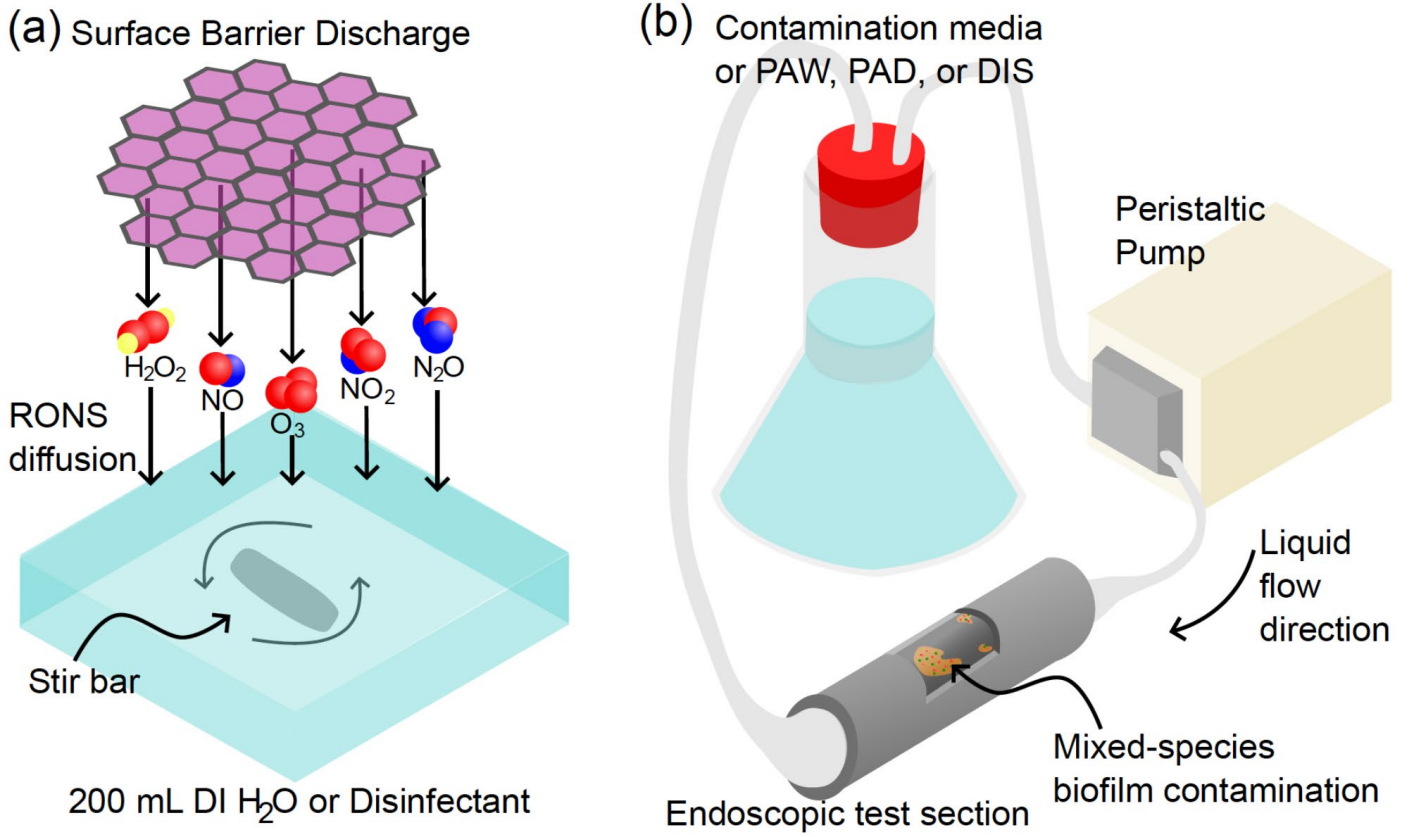
(**a**) Schematic diagram of the plasma system for activation of liquid. (**b**) Schematic diagram of the flow system used for contamination and decontamination of the endoscopic test pieces.

A low-temperature, surface barrier discharge (SBD) plasma source producing a thin layer of plasma within hexagonal gaps of a grounded mesh stainless steel electrode on the surface of a dielectric material was used. A 1 mm thick, 100 × 100 mm quartz plate was used as a dielectric barrier separating the mesh electrode from a high voltage copper plate electrode. This was positioned above 200 mL of each liquid (i.e., deionised water or pH buffered peracetic acid) with continuous stirring for 25 min and operated at a constant power of 30 W, allowing longer-lived reactive species to reach the liquid surface (Fig. 1(a)).

### PAL characterization

RONS were measured using spectrophotometric assays (SPECTROstar Nano, BMG LABTECH). Nitrite (NO_2_^−^) was measured at 548 nm following addition of Griess reagent (Supelco Ltd, MFCD01866819). Nitrate (NO_3_^−^) was measured at 420 nm based on interaction with sodium salicylate (Sigma-Aldrich Ltd, CAS 54–21 − 7) in a sulfuric acid medium after evaporation. Spectroscopy using TiOSO4 reagent in a sulfuric acid solution (Sigma-Aldrich Ltd, CAS 123334-00-9) at 410 nm was used to quantify hydrogen peroxide (H_2_O_2_). The pH was measured using a pH probe (Hanna Instruments 9813-6 with pH probe HI-1285-6).

### Contamination of endoscopic test pieces

As depicted in Fig. 1(b), a flow system connected to a peristaltic pump circulating at 100 mL/min was used to contaminate endoscope surrogate test pieces 10 cm in length, prepared from translucent polytetrafluoroethylene (Teflon) tubing with a 6.0 mm lumen diameter. Test pieces were filled with 200 mL of tryptic soy broth (TSB) containing 1% human serum and incubated at 37 ^o^C for 24 h to mimic exposure to bodily fluids and increase bacterial adherence potential. Subsequently, the system was contaminated with 10^6^ colony forming units (CFU) of a 200 mL culture containing 25% *Staphylococcus aureus USA300 JE2*, 25% *Staphylococcus epidermidis 1457*, 25% *Pseudomonas aeruginosa PA01* and 25% *Escherichia coli Bw25113*^27^. The contamination liquid was circulated through the test pieces for 45 min, followed by rinsing with distilled water and recirculation of the contamination liquid from the previous step. This procedure followed the established protocol for replicating endoscopic contamination with minor modifications, such as 1% human serum instead of 0.1%^27–29^. The system was left to cultivate for 24 h at 37^o^C.

### Comparison of disinfection methods

A pH-buffered peracetic acid (DIS: Olympus EndoAct/EndoDis, Olympus Surgical Technologies) prepared in a 1:1 ratio at a 2% concentration was compared with PAW. The plasma device was also used to activate the pH-buffered disinfectant, herein referred to as plasma activated disinfectant (PAD). Each method was circulated through the contaminated endoscopic test pieces in the flow system at 100 mL/min for 1, 3, and 5 min at room temperature. PAD concentrations of 0.5, 1 and 2% were also tested. The system was rinsed post disinfection, and the test pieces used for biofilm analysis.

### Analysis of regrowth and recolonization

Bacterial regrowth was tested by placing the disinfected test pieces in fresh TSB and incubating at 37^o^C, 200 rpm shaking for a further 24 h. Analysis of bacterial recolonization involved recirculating contamination fluid after a round of disinfection and incubating for 24 h at 37 ^o^C to assess the effect of treatment on bacterial reattachment. The total CFU remaining were determined using the Miles and Misra plating method.

### Surface characterization

The chemical composition of the endoscopic test pieces was determined by X-ray Photoelectron Spectroscopy (XPS) analysis. A TFA XPS spectrometer, produced by Physical Electronics Inc. operating under ultra-high vacuum (10 ^–7^ Pa) and equipped with a monochromated Al Kα X-ray source (1486.6 eV) was used. The take-off angle of the electron analyzer in the XPS spectrometer was 45° with respect to sample surface. The surface roughness and morphology of the endoscopic test pieces was assessed using Atomic Force Microscopy (AFM) (Solver PRO, NT-MDT, Russia). Silicon cantilevers with a typical resonant frequency of 240 kHz and a spring constant of 11.8 N/m were used to acquire images in semi-contact mode at room temperature under ambient conditions. The scanning rate was 1.5 Hz.

### Statistical analysis

All experiments were conducted using a minimum of 3 biological repeats and 3 technical repeats. Results are presented as mean ± standard deviation. Statistical analysis was performed using GraphPad Prism 10.2.3. One-way ANOVA was used with a Tukey’s multiple comparison to compare treatment groups for different concentrations of PAD (0%, 0.5%, 1%, and 2%). And to compare all groups (Control, PAW, DIS and PAD) for regrowth and recolonization. Allowing for comparison of means across multiple groups, while controlling for Type 1 error and identifying specific group differences. A p-value of ˂ 0.05 was considered as significant.

## Results and discussion

### PAL characteristics

**Fig. 2:**
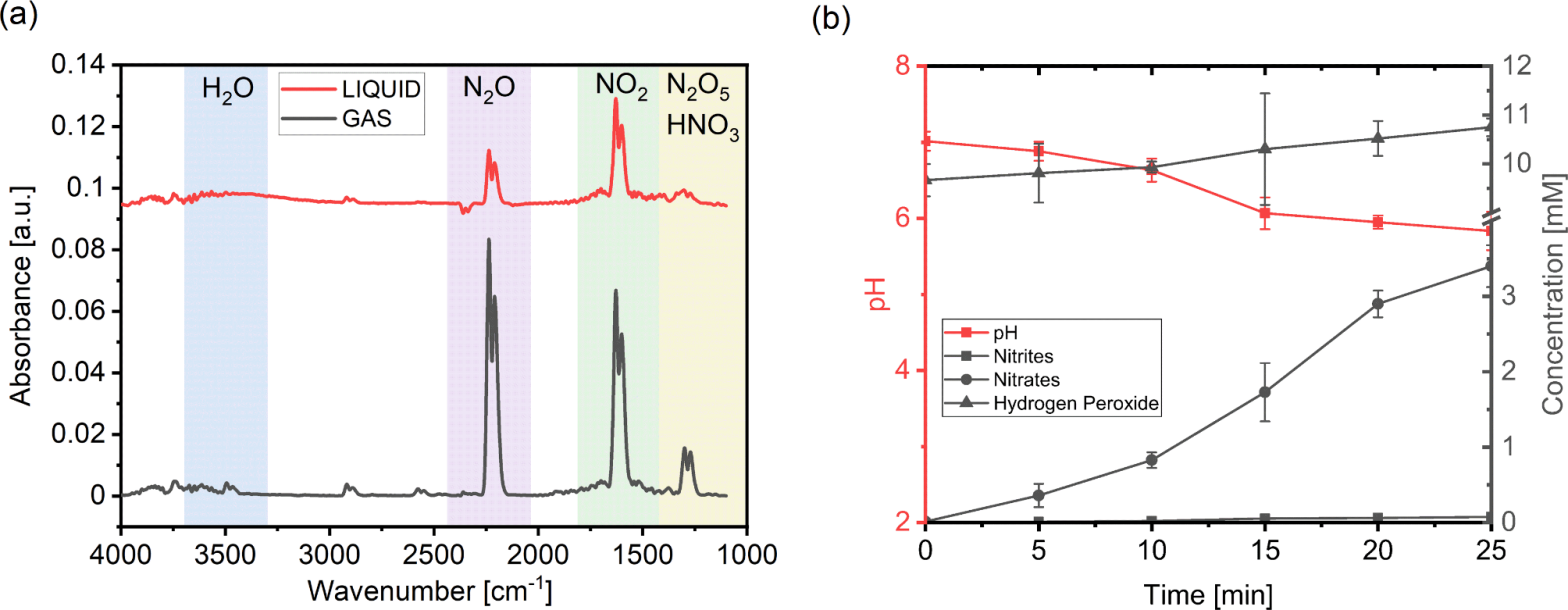
(**a**) FTIR absorbance spectra of the plasma effluent in the presence (red) and absence of liquid (grey) (**b**) Kinetic evolution of pH, nitrites, nitrates, and hydrogen peroxide within the plasma activated disinfectant (PAD) over a 25 min plasma activation period. Data presented show mean ± SD from 6 replicates.

In this study, PALs were explored for their efficacy in removal of mixed species biofilm contamination within the narrow lumen of endoscope working channels. The CAP system used for activation of the liquids produced a plasma with an afterglow dominated by reactive nitrogen species (RNS), as described in previous work^30^. The chemical composition of the resulting PAW has been previously detailed, therefore focus was placed on the evolution of the pH and chemical composition of the PAD over the 25 min plasma activation period. As displayed in Fig. 2(a), the absorbance of the afterglow obtained via FTIR spectroscopy shows the presence of longer-lived species, such as N_2_O, NO_2_, N_2_O_5_ and HNO_3_. This is a result of ionization, excitation, and dissociation reactions of O_2_, N_2_ and H_2_O between the electrodes ^24,31^. The longer-lived species reach the liquid via diffusion and convection and diffuse into the aqueous phase at the liquid interface to form NO_2_^−^, NO_3_^−^ and H_2_O_2_^24,31^. Evidence of the diffusion process occurring can be seen from the smaller FTIR peaks when liquid is present and the increase in NO_2_^−^ and NO_3_^−^ concentrations within the PAD solution over the 25 min activation period to final concentrations of 0.08 (± 0.003) mM and 3.40 (± 0.28) mM, respectively (Fig. 2(b)). The formation of NO_2_^−^ and nitrates NO_3_^−^within the liquid is described in the literature as the cause of the acidification of the solution^32,33^. A reduction in pH was observed in the PAD solution from 7.02 (± 0.13) to 5.83 (± 0.25), however activation of water can result in an acidic solution with a pH < 3^30,33,34^. It is suggested that the buffer present within the disinfectant solution prevents large reductions in the pH despite the presence of nitrites NO_2_^−^ and NO_3_^−^. H_2_O_2_ is present within the disinfectant solution at a concentration of 0.3% and continues to increase in concentration over the activation period from 9.67 (± 0.33) mM to a final concentration of 10.75 (± 0.18) mM. It has been described that H_2_O_2 _is reduced by ferrous iron to a reactive radical which causes damage to cellular and extracellular DNA resulting in cell mutations and disruption of the biofilm matrix^35,36^. Research into PAW has shown that the acidic solution possesses antibiofilm capabilities as a result of the reactive species present^35,37,38^. Hence, the presence of additional reactive species within the disinfectant is likely to increase its antibiofilm capabilities.

### Comparison of disinfection methods

**Fig. 3:**
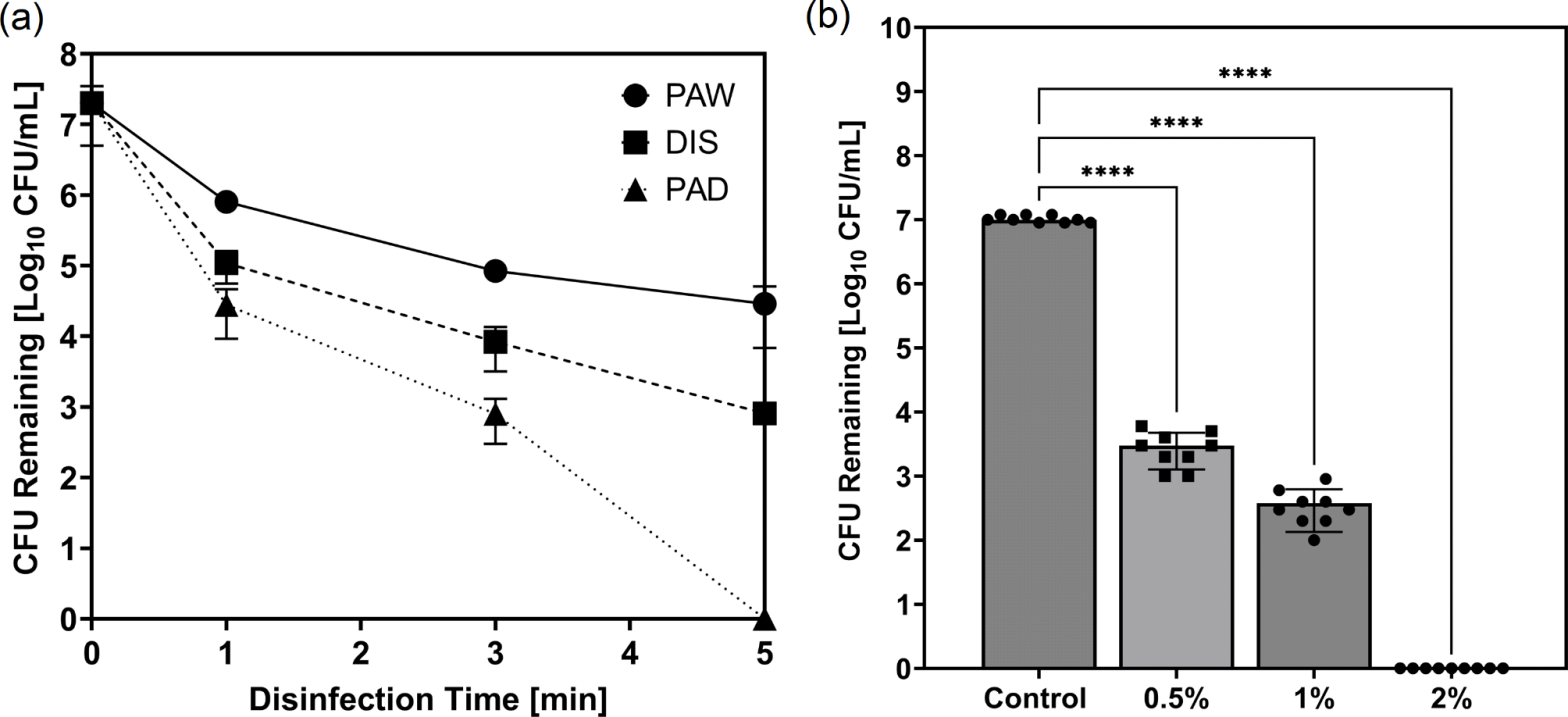
(**a**) Comparison of disinfection methods: PAW, DIS and PAD. (**b**) CFU remaining following disinfection treatment with PAD at concentrations of 0.5%, 1% and 2%. Data presented show mean ± SD from 9 replicates. **** *P* < 0.0001. Statistical analysis: one-way ANOVA with Tukey’s post-hoc test.

According to the Spaulding Classification, flexible endoscopes are semi-critical devices due to contact with mucous membranes or non-intact skin^9^. At the very minimum they should receive HLD, yielding a 6 log_10_ reduction, to prevent patient cross contamination^9^. This HLD encompasses all stages of endoscope reprocessing, not just the disinfection stage as explored here. Three disinfection methods were compared for their ability to remove mixed species biofilm contamination within endoscopic test pieces. Here it is shown that a commercially used pH buffered disinfectant (DIS) is capable of reducing mixed species biofilm contamination by a 4.39 log_10_ reduction factor, however PAD surpasses this performance reaching a 7.30 log_10_ reduction factor (Fig. 3(a)). PAW was the least effective with a log_10_ reduction factor of 2.84 in the CFU of mixed species biofilms treated for 5 min. PAD provides a level of disinfection surpassing the antimicrobial activity of PAW and the HLD required by the Spaulding Classification. Interestingly, a 3 min treatment with PAD resulted in a reduction by a factor of 4.40 in the CFU of biofilm contamination equalling the results seen by the commercially used disinfectant for 5 min treatment. As mentioned previously, peracetic acid is known to cause damage to endoscopes over time, therefore as results suggested PAD was more efficient than DIS, lower concentrations of disinfectant were activated using the plasma device. Figure 3(b) displays the CFU remaining following treatment with 0.5, 1 and 2% PAD solutions. A log_10_ reduction factor of 3.54 was seen for biofilms treated with 0.5% PAD. Treatment with a 1% PAD solution resulted in a log_10_ reduction factor of 4.44, and 2% again showed significant complete removal of culturable biofilm contamination reaching a log_10_ reduction factor of 7.00 (One way ANOVA: F(3, 32) = 549.0, *P* < 0.0001, R² = 0.9809, post hoc Tukey test: *P* < 0.0001). High inactivation rates matching or surpassing the current standard are also shown for PAD at lower disinfection times and lower disinfectant concentrations, indicating the possibility for shortening the overall time of the reprocessing cycle or reducing the concentration of harmful chemicals used to prevent damage.

### Effect of disinfection method on regrowth and recolonization

**Fig. 4:**
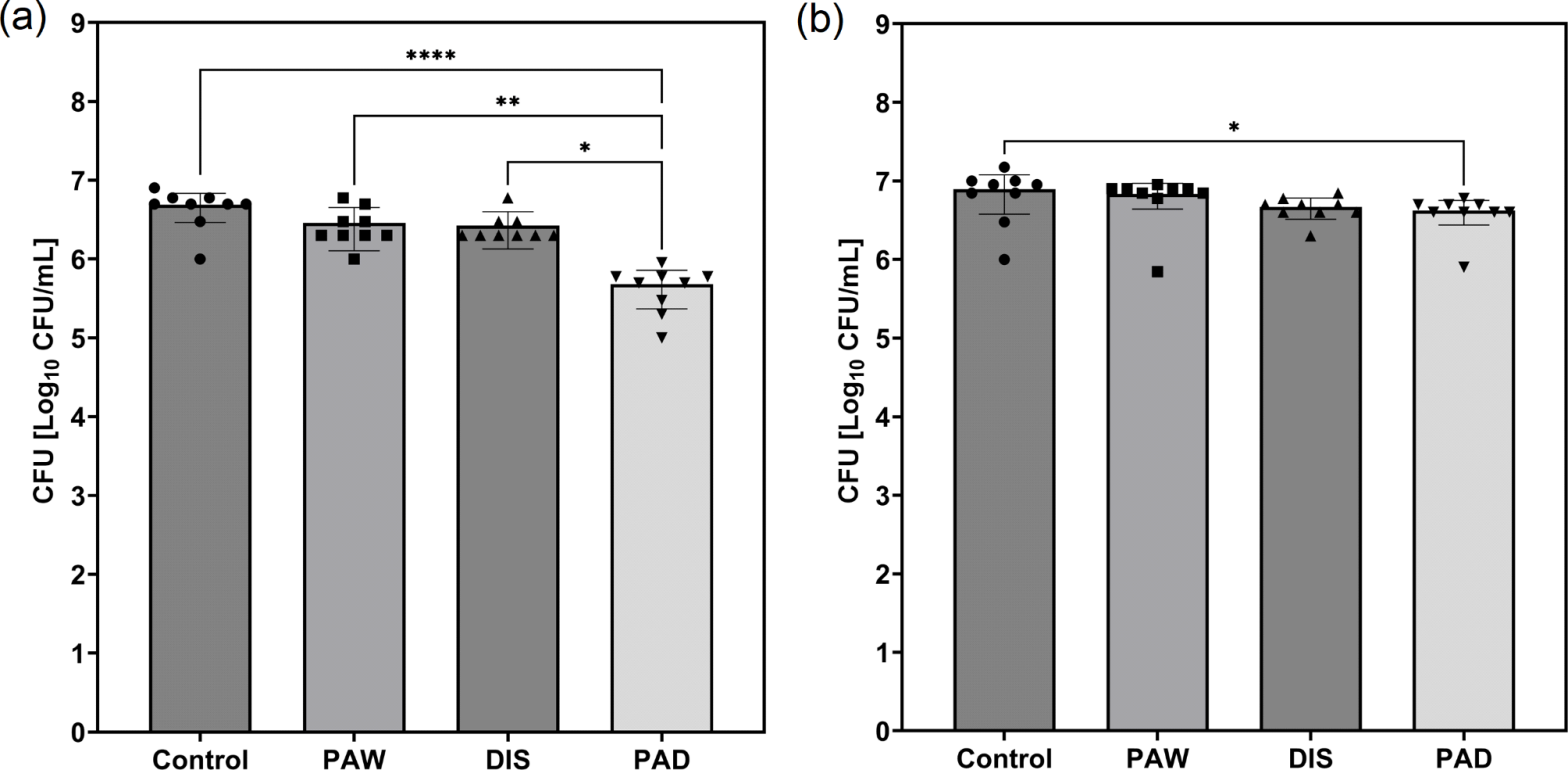
(**a**) Regrowth of biofilm contamination following disinfection with PAW, DIS and PAD. (**b**) Recolonization of the endoscopic test pieces following disinfection with PAW, DIS and PAD. The values are presented as the mean ± SD from 9 replicates. *P < 0.05, ** *P* < 0.001, ****P < 0.0001. Statistical analysis: one-way ANOVA with Tukey’s post-hoc test.

Regrowth and recolonization are important aspects of disinfection which are often overlooked. Endoscopic test pieces treated with each disinfection method were placed in fresh TSB and left for 24 h to assess regrowth. The endoscopic test pieces treated with PAW and DIS both had resulting average CFU counts not statistically different from the control, reaching 2.9 × 10^6^ and 2.7 × 10^6^ CFU/mL respectively (Fig. 4(a)). Most notably, the endoscopic test pieces treated with PAD showed significantly lower regrowth than all other cases with bacterial levels reaching a final concentration of 4.2 × 10^5^ CFU/mL (One way ANOVA: F(3, 32) = 14.15, *P* < 0.0001, R² = 0.5701, post hoc Tukey test: *P* < 0.0001, *p* = 0.0063 and *p*= 0.0145). The presence of regrowth following 5 min PAD disinfection indicates the presence of viable but nonculturable (VBNC) organisms after PAD disinfection. As only the disinfection stage of endoscope reprocessing was explored here, it is highly likely that any remaining VBNC organisms in the PAD condition would be removed. Another possibility for the results observed is false-negative culture results due to a lack of neutralizer post disinfection, as shown by Kawkman et al^39^. However, the improved efficiency compared to DIS remain valid as both were treated in the same manner. Regrowth is an important factor to consider as endoscopes can only be stored for a set amount of time before it is assumed that bacterial counts have reached unsafe levels and they must be reprocessed again. A systematic review by Schmelzer et al. found that storage time ranged from 2 to 56 days, and concluded endoscopes could be stored for 7 days but ongoing surveillance cultures were necessary^40^. Research has found that, despite recommendations of no longer than 7 days storage, reprocessed endoscopes often reach unsafe levels of bacterial contamination when left overnight or over the weekend^41^. It is suggested that the lower regrowth exhibited in test pieces treated with PAD could allow for longer safe storage times between reprocessing than with current disinfection methods.

Recolonization of the endoscopic test pieces following each disinfection method was explored due to evidence in the literature that some plasma disinfection methods can have changes to bacterial adherence as a result of surface modification^23,38,42^. Recolonization was not significantly different between the control, PAW and DIS treated test pieces (Fig. 4(b)). Endoscopic test pieces showed significantly reduced recolonization compared to control endoscopic test pieces after treatment with 5 min of PAD (One way ANOVA: F(3, 32) = 4.119, *P* = 0.0141, R² = 0.2786, post hoc Tukey test: *P* = 0.0248). As a result of these findings, surface analysis of the endoscopic test pieces was conducted after multiple rounds of each disinfection method to assess potential surface damage.

### Effect of PAL on surface characteristics

**Fig. 5:**
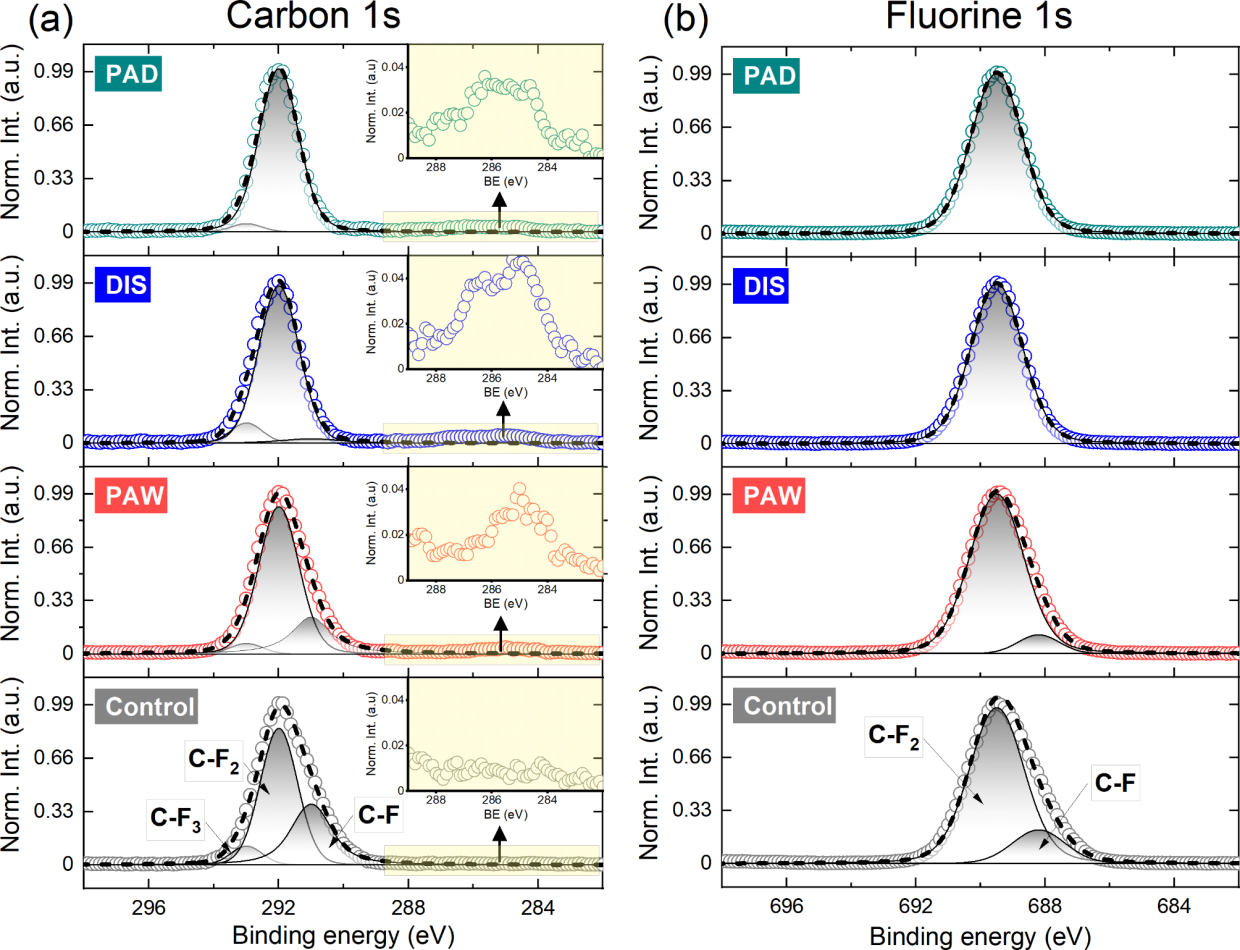
XPS analysis of the chemical composition of the endoscopic test pieces. Data presented shows high-resolution XPS spectra for carbon 1s (**a**) and fluorine 1s (**b**) with corresponding peak fittings.

Exploration of changes to the chemical composition of the endoscopic test piece surfaces were investigated to understand why PAD had significantly lower regrowth and recolonization. Analysis was carried out using XPS following multiple (5 × 5 min) rounds of each disinfection method. As shown in Fig. 5, endoscopic test pieces displayed a typical spectrum of Teflon with a strong carbon peak located at around 292 eV, and another intense peak at around 689.5 eV attributed to fluorine^43^. A detailed Gaussian/Lorentz deconvolution of the main peaks was performed and showed a relative increase in the CF_2_ peak area, and a relative decrease in C-F bonds following all disinfection methods. Peak areas are provided in detail in Supplementary Table S1. The C/F atomic ratio obtained for the endoscopic test pieces was 0.51, a typical atomic ratio for Teflon. All disinfection methods resulted in a minor increase in C/F atomic ratio compared to the control, 0.53, 0.55 and 0.57 for PAW, DIS and PAD, respectively. The results indicate all disinfection methods provoke a slight defluorination of the surface as a result of removal of C-F bonds. A study by Kim into the effects of argon and oxygen plasma treatments on polytetrafluoroethylene film also showed defluorination of the surface following exposure to plasma^44^. However, results provided here show that use of PALs cause vastly reduced changes to the chemical composition of the surface than the substantial surface modification seen within the study by Kim and similar studies^44–46^.

**Fig. 6:**
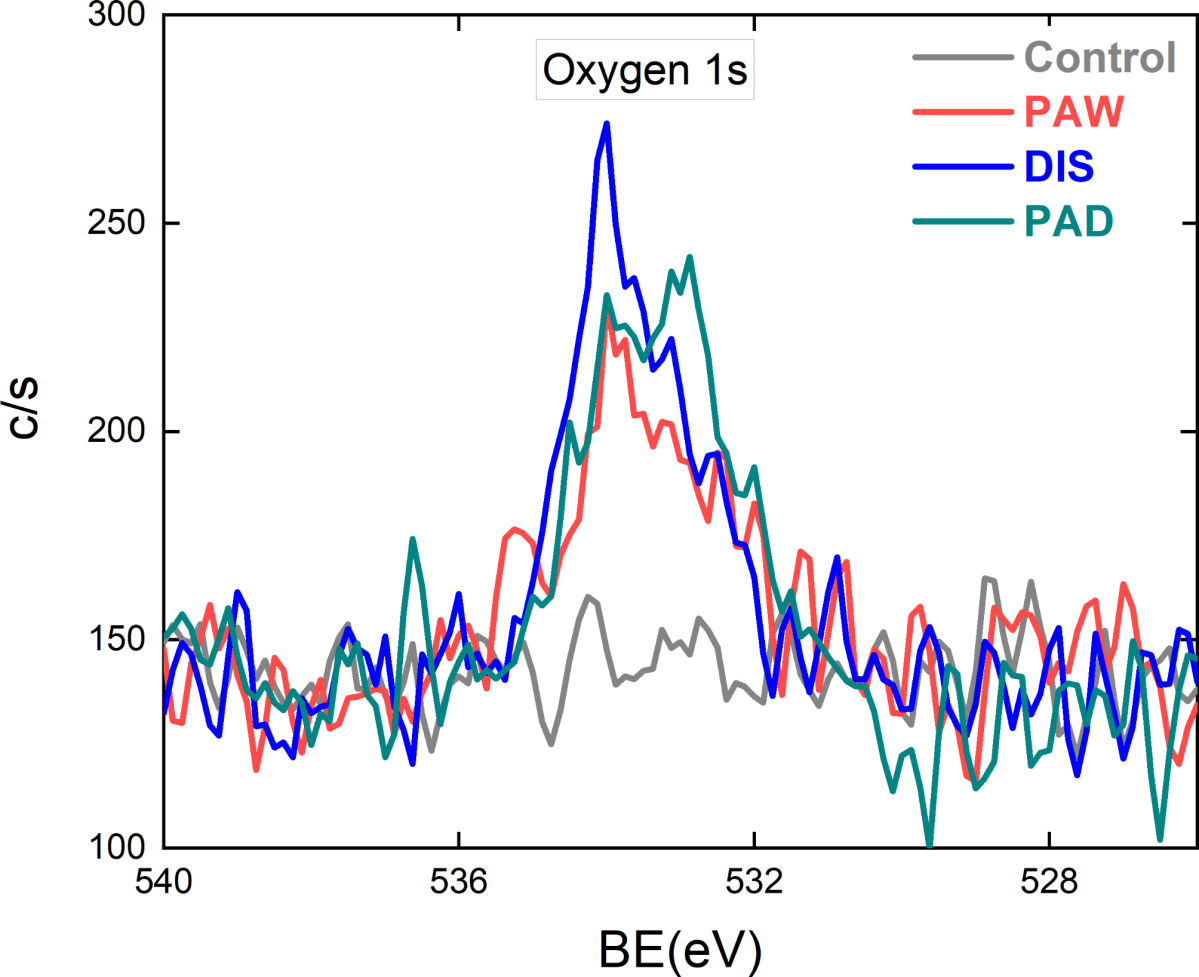
High-resolution region of oxygen (O 1s) for corresponding samples.

Figure 6 shows that all three disinfection methods resulted in a trace contribution of oxygen related peaks at around 534 eV. The atomic concentration of oxygen increased from 0.1 for the control to 0.8, 1.0, and 1.1 for PAW, DIS and PAD, respectively. This contribution is to be expected from these disinfection methods. A slight increase in C-O across all disinfection methods is shown in Fig. 5, which is a result of the carbon from weaker single bonded CF binding with the oxygen provided by the disinfection methods. The pH buffered peracetic acid used within this study is known to be a strong oxidant, with the oxidation potential of peracetic acid being higher than other disinfectants such as hydrogen peroxide or chlorine^47^. Oxidation as a result of plasma treatments is typical, but significantly more substantial with direct plasma treatments as opposed to PALs^44,48,49^. However, Teflon shows high resistance. The XPS results presented show that PAW resulted in the least changes to the surface of the endoscopic test pieces, however differences between DIS and PAD disinfection were minor, and it cannot be said that PAD disinfection would be more damaging. It is also important to note that these results indicate very minor surfaces changes that would be considered cosmetic changes and are expected as a result of exposure to any HLDs. ATR-FTIR was also used to explore any further changes in the surface characteristics of the endoscopic test pieces following multiple cycles of each disinfection method. As shown in Supplementary Figure S1, the absorbance spectra measurements are typical of Teflon, and demonstrate that endoscopic test pieces show no change as a result of all disinfection methods^50,51^. ATR-FTIR only provides snapshot information from a very small section of the Teflon tubing, hence multiple surface analysis methods were used to provide more detailed insight into any affects to the surface.

**Fig. 7:**
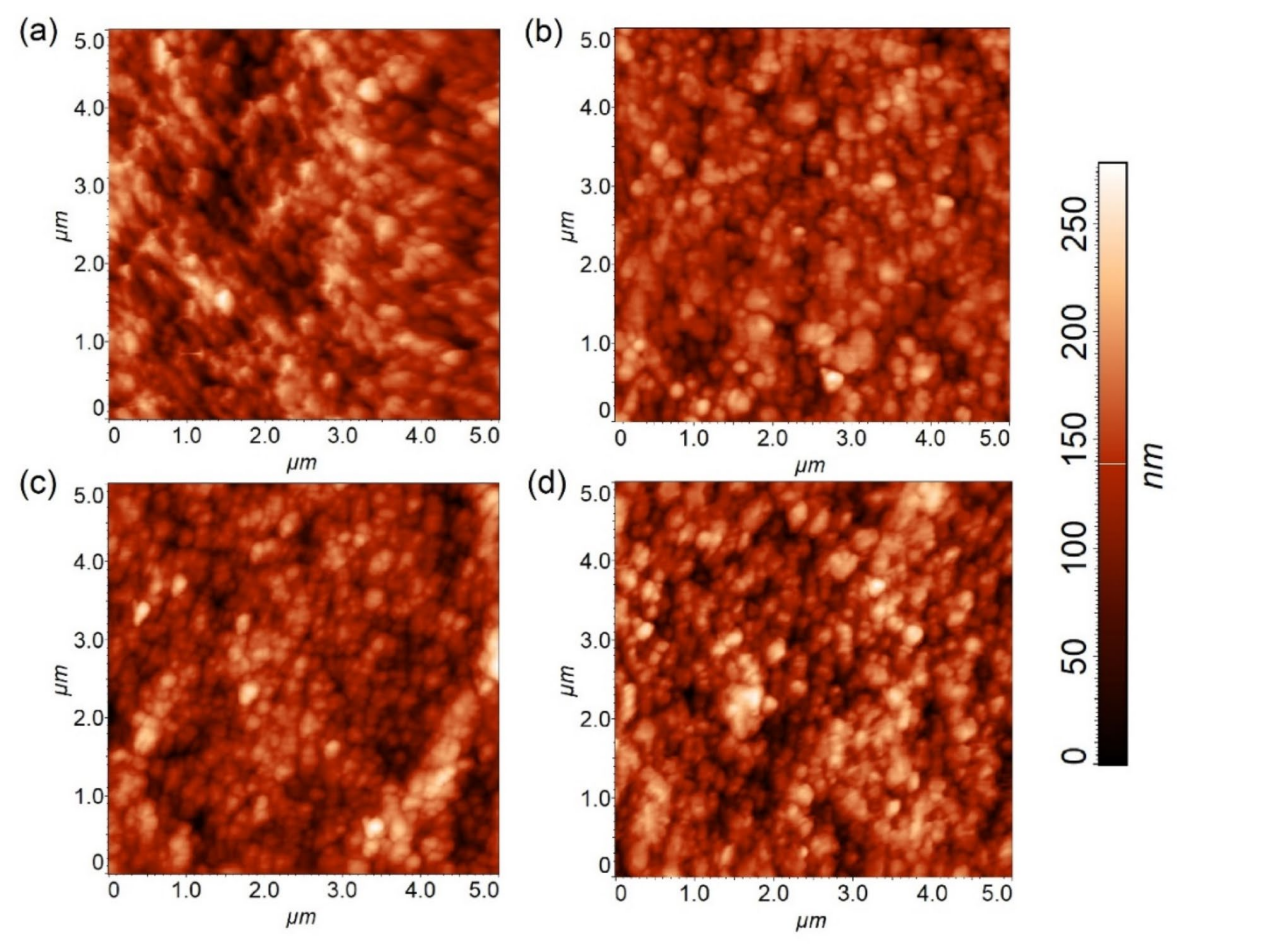
Representative two-dimensional morphological images of endoscopic test pieces before and after plasma treatment cycles: (**a**) control, (**b**) PAW, (**c**) DIS, and (**d**) PAD.

The influence of disinfection methods on the surface roughness and morphology of endoscopic test pieces was examined using AFM (Fig. 7). Visualisation of the morphology and calculation of the mean surface roughness (R_a_) was conducted using a minimum of three randomly selected areas on each endoscopic test piece. The calculated R_a_ for endoscopic test pieces exposed to multiple rounds of each disinfection method are shown in Supplementary Figure S2. Endoscopic test pieces had an R_a_of 21.46 (± 5.83) nm, 15.62 (± 2.24) nm, 21.98 (± 1.35), 19.18 (± 1.32) nm for control, PAW, DIS and PAD treated samples, respectively. No significant difference was found between the control and treated test pieces. Increases in surface roughness over time can result in increased bacterial adherence, decreased efficacy of HLD, and increased biofilm formation within the crevices and dips of the surface^52^. These issues have been highlighted by Bisset et al., who found a link between number of uses of an endoscope and frequency of microbial contamination detected, and presence of large amounts of biofilm in decommissioned endoscopes^53^. A study by Santos et al. conducted on endoscope working channel damage highlighted that damage caused by 500 passages of forceps in the endoscope resulted in a 3.2x increase in surface roughness^16^. While no increase in surface roughness was found within this study, it is important to note that only 5 rounds of disinfection were carried out and therefore, it is necessary to examine changes following more rounds of disinfection in a future study. However, the preliminary comparisons between the commercially available disinfectant and PAD presented here suggest that PAD would not cause significantly more changes over time than the current HLD option. Notably, findings indicate that PAD would not cause any more damage to the Teflon material used in the working channels of flexible than currently adopted methods.

## Conclusion

In conclusion, this study demonstrates that plasma activated disinfectant (PAD) can enhance the effectiveness of the endoscope disinfection stage by effectively removing biofilm contamination found with the narrow working channels. The results provide a strong basis for further exploration of integrating plasma activated liquids into an automated endoscope reprocessor for HLD. Not only does the activation of disinfectant result in significant removal of mixed species biofilm contamination, but also reduction in the concentration of chemicals used or the disinfection time provides levels of disinfection equal to that of a commercially used HLD. It is known that use of peracetic acid for disinfection causes damage to endoscopes over time, and a balance between efficiency and resulting damage must be struck. Minor surface changes were found, but no greater than those seen for peracetic acid. Regrowth and recolonization are a particular concern regarding endoscope disinfection, however test pieces treated with PAD had significantly lower regrowth and recolonization. Ultimately, it is clear that the developed approach could be a viable way to tackle the problem of failures in endoscope reprocessing as a result of biofilm contamination and warrants further exploration.

## Electronic supplementary material

Below is the link to the electronic supplementary material.

Supplementary Material 1

## Data Availability

The authors declare that the data supporting the findings of this study are available within the paper and its Supplementary Information files. Should any raw data files be needed they are available from the corresponding author upon reasonable request.
