# Supplementary material for: Evaluation of plasma activated liquids for the elimination of mixed species biofilms within endoscopic working channels
Source: Sci Rep. 2024 Nov 19;14:28593. doi: 10.1038/s41598-024-79276-4 (PMC11576748; doi:10.1038/s41598-024-79276-4)
Supplement: Supplementary file 1 — Supplementary Material 1 [file 41598_2024_79276_MOESM1_ESM.docx]

**Evaluation of plasma activated liquids for the elimination of mixed species biofilms within endoscopic working channels.**

Naomi Northage ^a, b^, Vasyl Shvalya ^b^, Martina Modic ^b^, Thorsten Juergens ^c^, Sascha Eschborn ^c^, Malcolm J. Horsburgh ^d^, James L. Walsh ^a, e, *^

^a^ Centre for Plasma Microbiology, Department of Electrical Engineering and Electronics, University of Liverpool, Liverpool, L69 3GJ, UK

^b^ Laboratory for Gaseous Electronics, Jožef Stefan Institute, Ljubljana 1000, Slovenia

^c^ R&D Endoscopy Reprocessing Systems, Olympus Surgical Technologies Europe, Olympus Winter & Ibe GmbH, Kuehnstraße 61, 22045 Hamburg, Germany

^d^ Infection Biology & Microbiomes, Institute of Infection, Veterinary and Ecological Sciences, University of Liverpool, Liverpool, L69 7BE, UK

^e^ York Plasma Institute, School of Physics, Engineering & technology, University of York, York, YO10 5DQ, UK

| **Sample** | **CF3 peak area (293 eV)** | **CF2 peak area (292 eV)** | **CF peak area (291 eV)** | **Carbon** | **Fluorine** | **Oxygen** | **C/F ratio** |
| --- | --- | --- | --- | --- | --- | --- | --- |
|  |  |  |  | **(at.%)** | **(at.%)** | **(at.%)** |  |
| Teflon | 0.15 | 1.26 | 0.75 | 33.8 | 66.1 | 0.1 | **0.51** |
| PAW-5x | 0.09 | 1.49 | 0.5 | 34.2 | 65 | 0.8 | **0.53** |
| DIS-5x | 0.16 | 1.58 | 0.08 | 35.3 | 63.9 | 1 | **0.55** |
| PAD-5x | 0.07 | 1.66 | <0.01 | 35.8 | 63.1 | 1.1 | **0.57** |

Supplementary Table S1. Relevant data after XPS analysis.


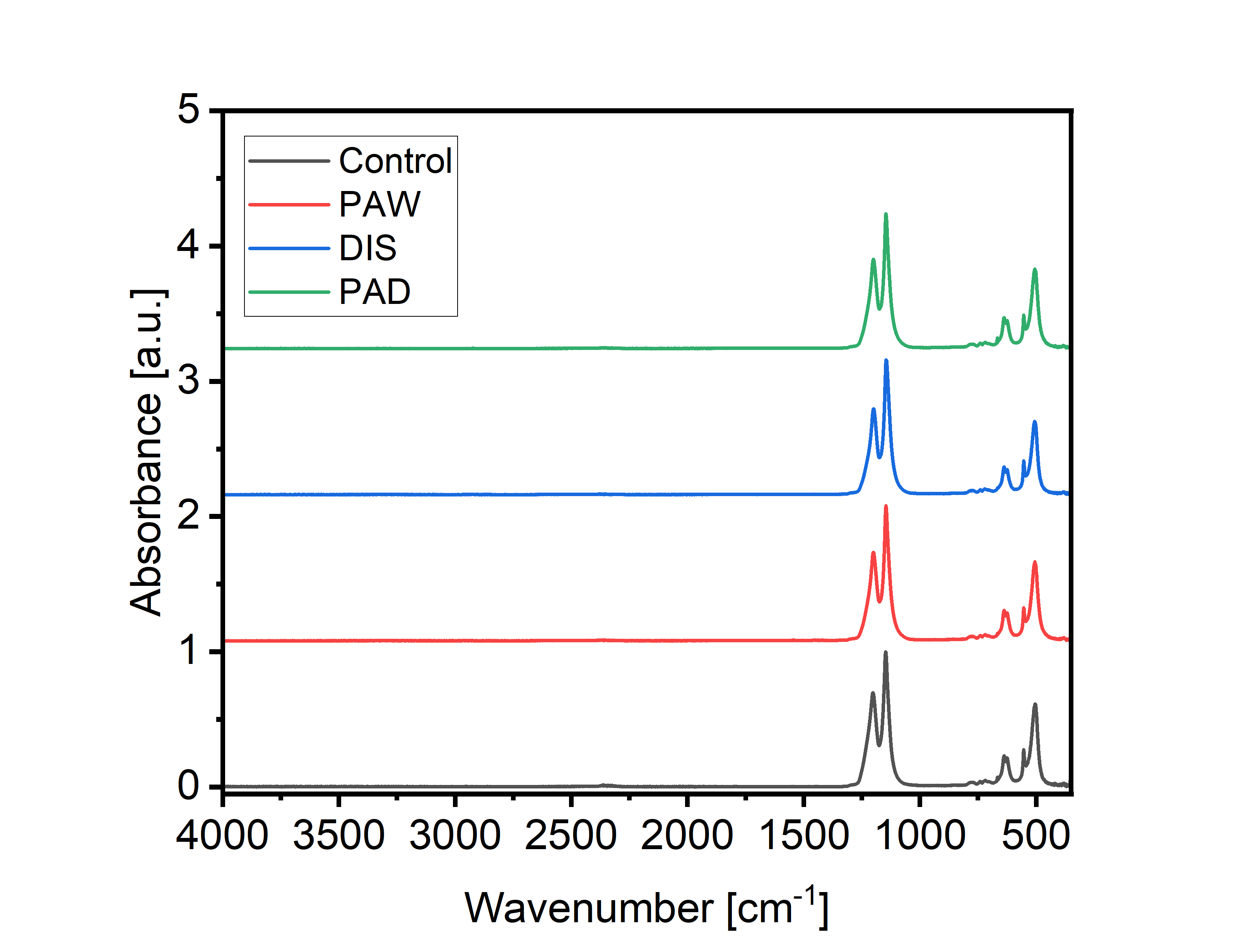


**Supplementary Figure S1.** ATR-FTIR absorption spectra of endoscopic test pieces obtained following 5x 5 min PAW, DIS and PAD treatments.


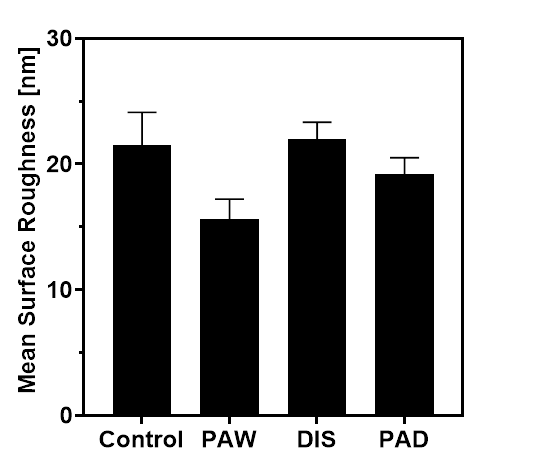


**Supplementary Figure S2.** Mean surface roughness for endoscopic test pieces treated with 5x 5 min cycles of PAW, PAD and DIS.
